# POLICY SERIES: TECHNOLOGY IN AGING: ARTIFICIAL INTELLIGENCE

**DOI:** 10.1093/geroni/igae098.0814

**Published:** 2024-12-31

**Authors:** Angela Perone, Brian Lindberg

**Affiliations:** Chair; Discussant

## Abstract

Artificial Intelligence (AI) is increasingly being used in aging research and gerontological education to analyze vast amounts of data and identify patterns that may offer insights into aging across the life course. By utilizing AI algorithms, researchers, practitioners, and educators can more efficiently study factors such as genetics, lifestyle, and social and environmental influences that contribute to aging. Machine learning techniques may also help predict the onset of age-related diseases and potential interventions. The incorporation of AI in aging research and gerontological education has the potential to revolutionize our understanding of aging and improve the quality of life for older people. Yet, it is not without ethical dilemmas that have sparked increased policy focus in recent months. This interactive session, organized by the GSA Public Policy Advisory Panel, is an interdisciplinary discussion addressing the use of AI in aging research and gerontological education and the related public policy implications. The panelists represent the six sections of GSA: ESPO, BS, BSS, SRPP, HS, and AGHE, along with the Minority Issues in Aging Advisory Panel and the Humanities, Arts, and Culture Gerontology Advisory Panel.

